# Preparation and Characterization of Cellulose/Silk Fibroin Composite Microparticles for Drug-Controlled Release Applications

**DOI:** 10.3390/polym16213020

**Published:** 2024-10-28

**Authors:** Suchai Tanisood, Yodthong Baimark, Prasong Srihanam

**Affiliations:** Biodegradable Polymers Research Unit, Department of Chemistry and Centre of Excellence for Innovation in Chemistry, Faculty of Science, Mahasarakham University, Mahasarakham 44150, Thailand; suchai.t@msu.ac.th (S.T.); yodthong.b@msu.ac.th (Y.B.)

**Keywords:** blue dextran, cattail, cellulose, microparticle, silk fibroin, water-in-oil

## Abstract

Microparticles derived from biomaterials are becoming increasingly popular for application in drug delivery systems. In this study, the water-in-oil (W/O) emulsification–diffusion method was used to create cellulose (C), silk fibroin (SF), and C/SF composite microparticles. We then observed the morphology of all obtained microparticles using scanning electron microscopy (SEM), evaluated their functional groups using attenuated total reflection–Fourier transform infrared spectroscopy (ATR-FTIR), and conducted thermogravimetric analysis using a thermogravimetric analyzer (TGA). SEM micrographs indicated that the native SF microparticles have the highest spherical shape with smooth surfaces. With blue dextran, the C microparticle was smaller than the native microparticle, while the drug-loaded SF microparticles were larger than the native microparticle. The morphological surfaces of the C/SF composite microparticles were varied in shape and surface depending on the C/SF ratio used. The spherical shape of the C/SF composite microparticle increased as the SF content increased. Furthermore, the size of the drug-loaded C/SF composite microparticles increased when the SF content gradually increased. The significant functional groups in the C and SF structures were identified based on the ATR-FTIR data, and a suggestion was made regarding the interaction between the functional groups of each polymer. When compared to both native polymers, the C/SF composite microparticles exhibit improved thermal stability. XRD patterns indicated that all prepared particles have crystalline structures and are directly affected by the released profile. The C/SF composite microparticle at a 1:3 ratio had the lowest drug release content, whereas the hydrophilicity of the C microparticle affected the highest drug release content. As a result, one crucial factor affecting the medication released from the microparticle is its structure stability. According to the obtained results, C, SF, and C/SF composite microparticles show promise as delivery systems for drugs with controlled release.

## 1. Introduction

Recently, the sustainable development goals (SDGs) policy has presented an attractive strategy to decrease the consumption of petroleum-based synthetic materials, as it is the main cause of environmental crises [1,2]. Utilizing biodegradable materials is one of the interesting ways to replace synthetic materials due to their eco-friendly, renewable nature, low cost, and wide availability [3,4]. The development of biodegradable materials has been performed for many applications, including food packaging [5,6,7], drug delivery systems [8], and biomedical products [9]. Cellulose (C) and silk fibroin (SF) are two of the most interesting biopolymers among the biodegradable materials that are regularly explored. Their sustainable sources and characteristics provide the most compelling motives.

The most prevalent biopolymer is C, a fiber of glucose units connected by β-1,4-glycosidic linkages throughout its molecular structure. When it comes to biomaterials, cellulose is superior to others in several ways, including its capacity to adapt mechanical properties, its biodegradability, and its thermal stability [10,11,12]. Furthermore, cellulose can be discovered in a wide range of organisms, such as marine life, plants, algae, and bacteria [13,14]. As a result, it may be referred to as a sustainable raw resource [15], which is also produced at a low cost. According to earlier studies, C has been created in a wide variety of forms based on its intended use [16,17,18]. These applications include food packaging [19,20], wastewater treatment [21], surface coating materials [22], and biomedicine [23].

The natural protein-based polymer, identified as silk, is spun into fibers by silkworms to generate silk cocoons. The two major components of silk fibers are fibrous SF and globular silk sericin (SE). The glue-like protein SE covers the main fiber SF [24,25]. The SE is typically discarded as a degradable waste material during the degumming procedure at the end of the silk yarn production process. Because of its biodegradable and biocompatible characteristics, SF is taken into consideration for potential construction in the biomaterial category [26]. SF-based devices have drawn a lot of interest for a variety of applications [27,28]. Additionally, SF has been processed to create several bioactive scaffolds [29].

Microparticles made from biopolymers have garnered attention in earlier research as appealing building blocks for possible devices in a variety of domains [30], particularly as drug carriers for delivery systems. Since it can be used to encapsulate medications or other active substances before being injected into the body’s target organs through a vascular vein, high encapsulation efficiency is a desirable feature that has drawn the attention of numerous researchers. Because of their clearly established model for degradation and release profile, biodegradable microparticles are frequently targeted for use as controlled-release vehicles [31,32,33]. There have been several reported methods for producing microparticles [34]. Each method has its own distinct advantages. We have developed microparticles from various biopolymers at our research unit by adopting the water-in-oil (W/O) emulsification–diffusion technique. We have also studied and reported on the optimal conditions and properties of SF microparticles, both with and without additional biopolymers [32,33,35]. Furthermore, our earlier work also produced cellulose microparticles [36]. Nevertheless, there are other aspects that need to be improved, and the cellulose microparticles that were produced were not up to the requirements of the perfect protocol. In addition, SF-mixed cellulose microparticles have never been developed in previous research. With that in consideration, the purpose of this study is to prepare the cellulose (C), SF, and C/SF composite microparticles by employing the water-in-oil (W/O) emulsification–diffusion method. The stirring rate, volume of C, SF, and C/SF, and W/O volume ratios were recorded for gathering data. The SF solution in this work was made by dissolving *B. mori* silk cocoons in a tertiary mixture solution of CaCl_2_:C_2_H_5_OH:H_2_O (1:2:8 by mol). Cattail raw materials were chemically treated to provide cellulose. It was established how different parameters affected the microparticles’ size, shape, and conformational change. Additionally, the solubility of the microparticles was examined and discussed.

## 2. Materials and Methods

### 2.1. Materials

The cocoons of *B. mori* silk were provided by the Silk Innovation Center at Mahasarakham University, located in the Khamriang sub-district of Kantharawichai, Maha Sarakham, Thailand, and the cattail (*Typha angustifolia* L.) trees were gathered from a pond. The cattail tree was chopped off at 10 cm from the rhizome and 15 cm from the tip. Tap water was then used to remove some dirt, and the tree was left to dry at room temperature. The samples were crushed and stored in plastic bags once they had dried. We obtained ethanol (C_2_H_5_OH), sodium carbonate (Na_2_CO_3_), and sodium chloride (NaCl) from the Merck KGaA company (Darmstadt, Germany) and Ajax Finechem Pty Ltd. (Auckland, New Zealand). Ethyl acetate (CH_3_COOC_2_H_5_), sodium hypochlorite (NaClO) and sulfuric acid (H_2_SO_4_) were purchased from Merck Life Science Private Ltd. (Maharashtra, India). Before use, none of the reagent-grade chemicals used in this study required additional purification.

### 2.2. Extraction of Cellulose

The gathered cattail samples were sequent cleaned twice with deionized water after an hour-long immersion in ethanol before drying at 80 °C for two hours. They were then weighed to determine their exact weight. The step of C extraction was followed with the previous report [36]. The 10 g samples were boiled in 100 mL of 4% NaOH for four hours. The solid samples were then separated by filtration and washed with distilled water to reach a neutral pH. To bleach the neutral samples, 2% (*v*/*v*) sodium hypochlorite (NaClO) was added and heated to 80 °C for two hours. Afterward, the reaction mixture was centrifuged to extract the residue. The reaction mixture was then hydrolyzed with 5% H_2_SO_4_ at 50 °C for three hours. The resulting mixture was then filtered and washed with distilled water to reach a neutral pH. Finally, C was obtained and stored in a refrigerator until further processing.

### 2.3. Preparation of SF Solution

The SF was derived from Thai silk *B. mori* cocoons (Nang Lai variety). After being gathered, the cocoons were cleaned and then chopped into small segments. To eliminate the SE, they were then boiled twice in a 0.5% (*w*/*v*) Na_2_CO_3_ solution for 30 min each time at 100 °C. The degummed silk samples were then rinsed with distilled water until the pH level was neutral to obtain the SF. Afterward, the SF was dissolved for 60 min at 75 °C with continuous stirring using a tertiary solvent system that consists of CaCl_2_:C_2_H_5_OH:H_2_O (1:2:8 by mol). Using a dialysis membrane (MW cut off 10 kDa, Thermo Fisher Scientific Inc., Waltham, MA, USA), the hydrolysate SF was dialyzed against distilled water for three days to exclude any salt. The concentration of the SF solution was then calculated, and distilled water was added to dilute it to 2% (*w*/*v*).

### 2.4. Preparation of C, SF, and C/SF Blend Microparticles

In this work, all the microparticles were produced using the water-in-oil (W/O) emulsification–diffusion method [30]. The C and SF solutions were used as the water phase, and ethyl acetate was used as the oil phase. The form, size, and characteristics were influenced by many parameters, including the volume, concentration, and rate of stirring of the polymers, as well as W/O ratios that were examined. The stirring rate was set up in the range of 600–800 rpm, and concentrations of the polymers were tried out at 0.5–1.5% (*w*/*v*). In the preparation step, the oil phase, ethyl acetate, contained in a container, was stirred on the magnetic stirrer apparatus. A suitable volume of each polymer solution was then gradually added to the solvent dropwise while stirring was maintained for 30 min. The C/SF blend microparticles were also constructed using the same method. Using constant conditions, a desirable SF volume and concentration have been identified for blending with the C solution. The different C/SF mixed ratios of 3/1, 1/1, and 1/3 (*v*/*v*) were prepared before use and stirred for 30 min to achieve homogenous solutions. To prevent the solvent from evaporating throughout the emulsification and diffusion processes, aluminum foil was placed over the beaker. Afterward, the particles were gathered by centrifugation, the solvent was completely evaporated, and they were dried at room temperature in a vacuum oven.

### 2.5. Preparation of Drug-Loaded Microparticles

The W/O emulsion solvent diffusion method was also used to prepare microparticles containing water-soluble drug models to observe the drug release profile. For ten minutes, the C/SF aqueous solutions were stirred magnetically. A native C and SF solution and 1.0 mL of a C/SF blend solution containing blue dextran (0.0025 g/mL) were gradually added dropwise to 100 mL of ethyl acetate while being magnetically stirred at 700 rpm for an hour. The drug-loaded microparticles were separated by centrifugation and then left in a vacuum oven to dry at room temperature.

### 2.6. An Analysis of the Microparticles’ Characters

#### 2.6.1. Morphology Observation

A scanning electron microscope (SEM) was used to investigate the morphology of each created microparticle. An aluminum stub was used for each type of microparticle. All examined microparticle surfaces were subjected to an electron-exciting Au sputter coating before being examined under a 15 kV.

#### 2.6.2. Analysis of Functional Group

The functional groups of the produced microparticles were examined using an attenuated total reflectance (ATR) accessory on a Fourier transform infrared (FTIR) spectrometer (Perkin Elmer-Spectrum Gx, USA). The results of the ATR-FTIR spectrum were obtained by using 32 scans and a range between 4000–400 cm^−1^ at a spectral resolution of 4 cm^−1^. This process was managed by using air as the reference.

#### 2.6.3. Thermal Stability

The thermal stability of the constructed microparticles was investigated using a thermogravimetric analyzer (TGA) (SDTQ600, TA-Instrument Co., Ltd., New Castle, DE, USA). The microparticles were placed inside an aluminum pan and heated at a fixed rate of 20 °C per minute between 50 and 800 °C. The procedure was carried out in a nitrogen-filled atmosphere. There was numerous weight losses documented over time.

#### 2.6.4. Crystallinity Analysis

To describe the crystallinity patterns of the prepared microparticles, X-ray diffraction (XRD, Bruker D8 advance, Germany, with Cu Kα, λ = 1.5406 Å, 40 kV, and 40 mA) was utilized. From 2θ = 5° to 60°, the diffraction angle varied with a step size of 0.02°/s.

#### 2.6.5. Drug Release Analysis

At room temperature, the mixed microparticles of the C, SF, and C/SF composite with blue dextran were submerged in a buffered saline, pH 7.4, while being shaken. A total of 2.5 mL of water was collected after the intervals of 1, 2, 3, 4, 8, 16, 24, and 48 h. The absorbance at 640 nm was then measured after the collected volume was replaced with the same fresh volume of water. By comparing the concentration of blue dextran released from the microparticles to a standard curve, the concentration was estimated.

## 3. Results and Discussion

### 3.1. Morphological Observation

#### 3.1.1. Cellulose Microparticles

**Figure 1:**
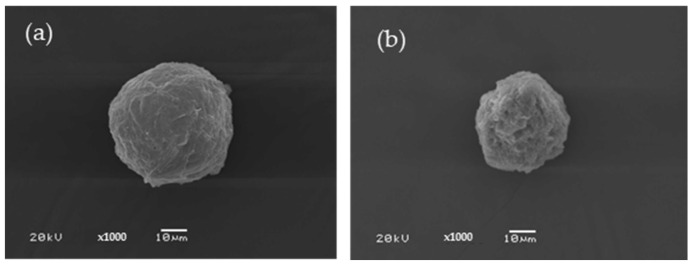
SEM micrographs of C microparticle (**a**) and blue dextran-loaded C microparticle (**b**).

The cellulose content extracted from cattail trees was 17.88 ± 0.61%. This amount was in the range consistent with previous reports [17,37]. The method for producing biopolymer microparticles using water-in-oil emulsion–diffusion has been published previously by our research group. A few variables, including the volume of each polymer, the speed of stirring, the ratio of the water (W) phase to the oil (O), and surfactants and cross-linking agents, all had an influence on the success of the microparticle production process [27,31,34]. The spherical shape of the microparticles is appropriate for application as a drug delivery device since it allows them to be easily and promptly transported throughout bodily fluid. Additionally, the spherical form has direction balance and might hold components that can be released in any direction [32,35]. Figure 1a illustrates the morphology of the 2.5% cellulose microparticles that were created. The prepared microparticles have a size in the range of 60–700 μm, with the largest distribution being 60–100 μm, followed by 200–500 μm. The results clearly illustrate that the manufactured microparticles have a somewhat complete appearance. It is possible to create completely spherical microparticles under these conditions. The C microparticles have a smooth surface and are packed closely together by chemical bond formation [38]. However, it was found that the particles’ surfaces are significantly wrinkled. Water evaporation while drying was thought to be the cause of this [39]. At low magnification, it was found that the particles were not dispersed equally. Naturally, cellulose is a semicrystalline fiber. Our previous work indicated that the cellulose microparticles have crystalline parts higher than the amorphous ones [36].

A hydrophilic substance called blue dextran was selected to investigate the effects of the C microparticles on drug encapsulation. Figure 1b shows the morphological outcomes of the blue dextran-containing cellulose microparticles. The findings showed that although the microparticles were fully formed, they were not as spherical as desired. The microparticles have an angular form in general. In comparison to the native C microparticles, blue dextran-loaded C microparticles have smaller sizes, with the largest distribution in the ranges of 45–80 μm and 200–400 μm. Since blue dextran is water-soluble, adding it might increase the polarity of the solution, which will interrupt the spherical form. Therefore, the spinning rate used for the microparticle construction decreased from 700 to 600 rpm. 

#### 3.1.2. Silk Fibroin Microparticles

**Figure 2:**
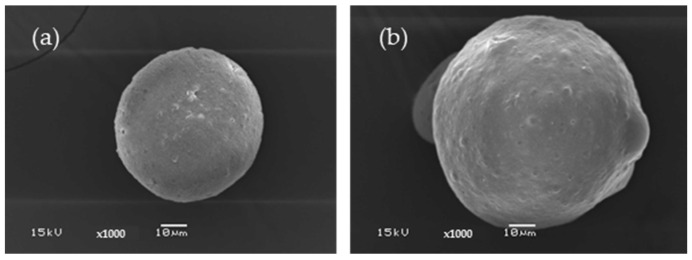
SEM micrographs of SF microparticle (**a**) and blue dextran-loaded SF microparticle (**b**).

Figure 2a shows a SEM micrograph of the SF microparticles. The microparticles have a spherical shape. The surface of the SF microparticle is smooth and has a solid texture. At low magnification, the prepared SF microparticles had different sizes; some particles have incomplete formation and are hollow. It also has a flat, elongated shape resembling an oval. When compared with C microparticles, SF microparticles are approximately 2–2.5 times smaller, ranging from 25 to 45 μm, and are comparable. Figure 2b displays the SF microparticles loaded with blue dextran. The outcome showed that the SF loaded with the drug could be formed into precisely formed spherical microparticles. Moreover, the drug-loaded SF microparticles showed about a twofold increase in size (45–80 μm) compared to the native SF. When considered in detail, it was found that the microparticles had a rough surface with irregular form. This is because the polar components of blue dextran may rapidly evaporate from the matrix. The less polar SF, on the other hand, attaches itself to the non-polar portion of blue dextran and does not evaporate. The microparticles’ spherical form is mostly due to the binding of SF. The polarity of a molecule, whether high or low, plays a significant role in the shape of the particles that are obtained. Furthermore, it has been observed that the stirring speed affects particle shape and size [34,35].

#### 3.1.3. C/SF Composite Microparticles

**Figure 3:**
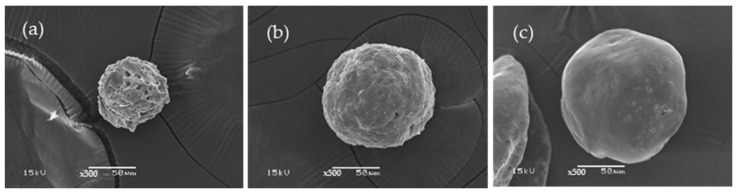
SEM micrographs of C/SF composite microparticles at 3:1 (**a**), 1:1 (**b**), and 1:3 (**c**) ratios.

**Figure 4:**
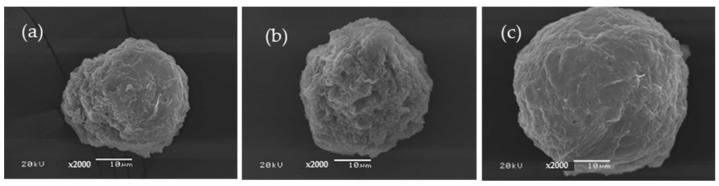
SEM micrographs of C/SF composite microparticles at 3:1 (**a**), 1:1 (**b**), and 1:3 (**c**) ratios, mixed with blue dextran.

The C/SF composite microparticles were prepared using 2.5% C and 2% SF with different volume ratios. As seen in Figure 3, the results showed that the best ratio used for the preparation of the composite microparticles was 1:3 (Figure 3c) since the obtained microparticles have a spherical shape with a smooth surface. The composite microparticles have a size in the range of 70–80, 120–130, and 155–165 μm for the blend ratios of 3:1, 1:1, and 1:3. In contrast to the SF microparticles, the obtained microparticles were discovered to not be spherical. Additionally, there were some indentations on the surface that went inside, and there were a few tiny holes. It is believed that the polar components of the microparticle evaporated during drying, resulting in these holes [19,40]. The two polymers, which are not homogenous mixes, are assumed to have separated, leading to the indentations into the microparticle. High concentrations of salt break down the peptide bonds that bind the various amino acids that make up SF, a fibrous protein. Achieving microparticles depends on the peptide chain’s size as well as the conditions under which it is prepared. A homopolysaccharide of glucose units connected by β-1,4-glycosidic bonds forms cellulose, another fibrous substance. Since the extraction process involves a few parameters, it is extremely challenging to regulate the size and amount of the C that is extracted. In this work, we proposed that the important factors for particle results are the size and aspect ratio of the C and SF. The forces and times between molecules that cause polar solutions to evaporate and spherical particles to form are different. The average size of the C/SF composite microparticles has decreased to 32, 35, and 45 μm for the blend ratios of 3:1, 1:1, and 1:3, respectively, with blue dextran (Figure 4). The findings demonstrated that blue dextran, a hydrophilic medication, could bind firmly to cellulose and cause a size decrease like that seen in the native C microparticles (Figure 1).

### 3.2. FTIR Spectra of Microparticles

**Figure 5:**
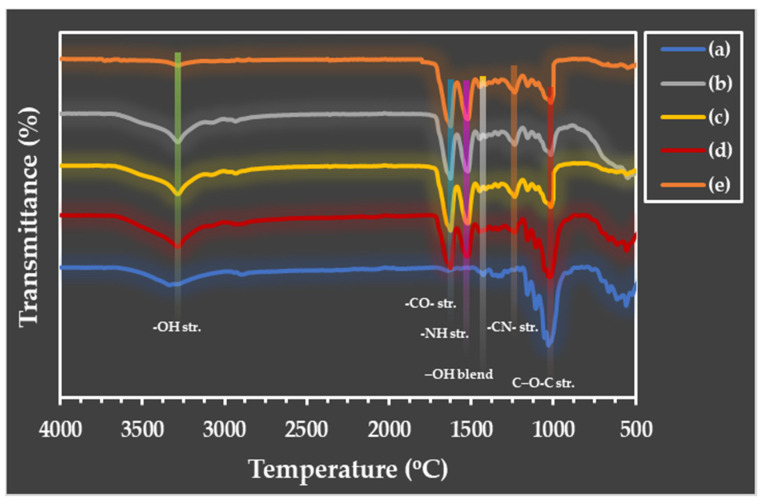
ATR-FTIR spectra of microparticles: native SF (a), C/SF composites at 1:3 (b), 1:1 (c), and 3:1 (*v*/*v*) ratios (d), and native cellulose (e).

The ATR-FTIR spectroscopy was used to investigate the functional groups of the generated microparticles, as illustrated in Figure 5. Significant functional groups of the C microparticle (Figure 5a) are seriously considered at the hydroxyl group’s absorption location, which is at 3352 cm^−1^ (-OH stretching). The carbonyl ester group (C=O stretching) of hemicellulose is represented by the absorption peak at 1635 cm^−1^, while the methyl group (-CH_2_ stretching) is positioned at approximately 2850–2950 cm^−1^. The absorption peaks of lignin can be found at 1300 cm^−1^ and 1014 cm^−1^, which correspond to the asymmetric location of the glucose units and C-O-C groups joined by the β-1,4 glycosidic linkages at 900 cm^−1^ [17,41,42].

The functional groups’ absorption peaks in the protein structure are displayed in Figure 5e. The peptide bond, or R-COONH-R, is where amino acids are bonded to one another. The locations of the three different amide types, including amide I, are found in the absorption range of 1700–1600 cm^−1^. The carbonyl group (-CO-) is associated with this peak; amide II can be seen in the absorption region range of 1600–1500 cm^−1^. This absorption range is associated with the amide III, methyl group (-CH), and amine group (-NH). An absorption region appears at approximately 1300–1150 cm^−1^. Absorption in this range involves groups such as -NH in the plane, -C-C-, and -CO- in the straight line, and the absorption peak is approximately 1042 cm^−1^ [43,44].

Table 1 summarizes the data on the maximums of the adsorption positions in the FTIR spectra of the SF/C composite microparticles mixed at various ratios. The SF microparticles have a β-pleated sheet structure at both the amide I and II positions. This structure makes the particles hard, brittle, and easy to break. Cellulose exhibits strong absorption at the hydroxy group (-OH) position. This group has a strong polarity, resulting in a soft and flexible structure. Considering the composite microparticles, the light absorption value shifted and changed in a way that improved the structure’s flexibility while maintaining its strength. Because of the higher concentration of silk fibroin, the microparticles retain a β-sheet structure at the amide I and II positions [45]. This finding demonstrates the compatibility of cellulose and silk fiber and the formation of interactions through the functional groups present in the structures of each polymer, including hydrophobic interactions, van der Waals forces, and hydrogen bonds [46].

### 3.3. Thermogravimetric Analysis

**Figure 6:**
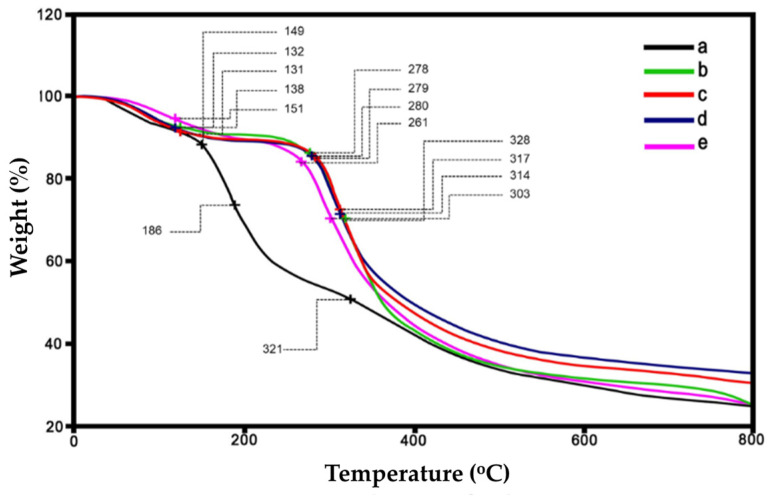
TG thermograms of microparticles: native cellulose (a), C/SF composites at 3:1 (b), 1:1 (c), and 1:3 (*v*/*v*) ratios (d), and native SF (e).

**Figure 7:**
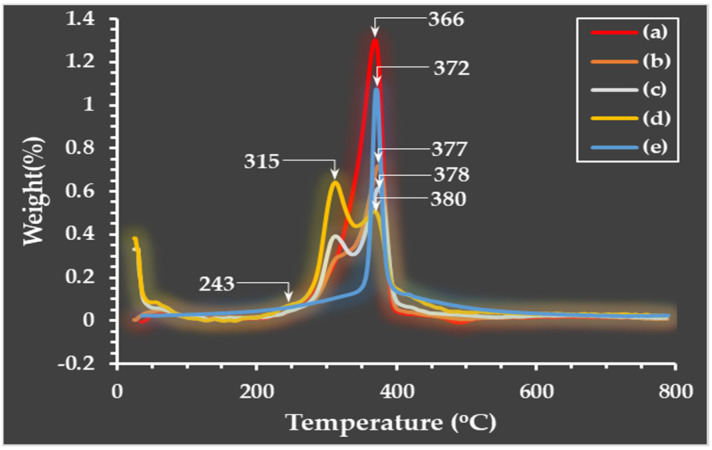
DTG curves of microparticles: native cellulose (a), C/SF composites at 1:3 (b), 1:1 (c), and 3:1 (*v*/*v*) ratios (d), and native SF (e).

A weight loss and the maximum decomposition temperature (T*_d, max_*) of the prepared microparticles across 0–800 °C were investigated using a thermogravimetric analyzer (TGA). According to the TG thermograms in Figure 6, the decomposition of microparticles occurred in at least three stages. These stages are clarified by the DTG curves exhibited in Figure 7. Small peaks indicate that water evaporation [34] from the microparticles occurred at low temperatures (60–80 °C), which was the cause of the initial decomposition. This suggested that the moisture content remained low in the microparticles. The second stages of decomposition were observed at 260–300 °C, which was dominantly revealed in the SF/C composite microparticles; the decomposition temperature of protein is in this region [27,32,34]. The results showed that the C/SF composite does not have a homogeneous texture compared to the native SF. The third stage (between 300 and 400 °C) dealt with the breakdown of silk fibroin [27,32] and cellulose [47]. Compared to native polymers, the charred residue of the composite microparticles is slightly higher at the end temperature test. Of the composite microparticles, the C/SF with a 1:3 ratio had the highest value, approximately 35%. The ratios of 1:1 and 3:1 had burned residues of 30% and 28%, respectively. These charred residues were no longer degraded in the range of the tested temperatures during heating. Moreover, the native C microparticle had a greater degradation at the beginning of the heating temperature. This is due to the hydrophilic functional group in the C structure being broken and evaporating in high water content. Table 2 provides an overview of each microparticle’s T*_d, max_*. Because of the establishment of robust intermolecular interactions between C and SF, the decomposition temperature of the C/SF composite microparticles increased and continued to increase gradually by increasing the SF content. Therefore, this result indicates that C and SF are joined together via the mentioned interaction, improving their thermal stability [48,49].

### 3.4. Crystallinity Patterns

**Figure 8:**
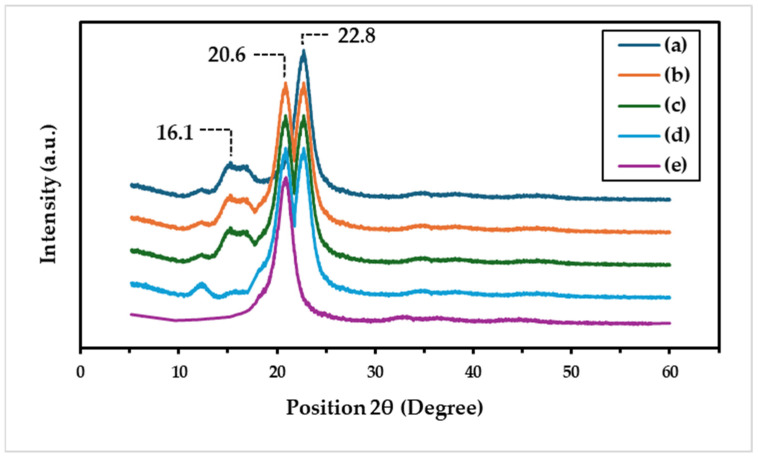
XRD of microparticles: native cellulose (a) C/SF composites at 1:3 (b), 1:1 (c), and 3:1 (*v*/*v*) ratios (d), and native SF (e).

The crystallinity of all prepared microparticles was examined using X-ray diffraction (XRD). As shown in Figure 8a, the XRD pattern of the native C microparticle showed diffraction peaks at around 14.5°, 16.5°, and 22.5°, which matched the cellulose I pattern [50]. The native SF microparticle revealed the main diffraction peaks at 20.6°, which were a crystalline structure of the SF [51]. The C/SF composite at different ratios (Figure 8b–d) still exhibited these dominant peaks with a slightly stronger crystalline structure. This confirmed that the structure of the C and SF microparticles was crystalline and enhanced the strength of the microparticle texture.

### 3.5. Releasing of Blue Dextran from Microparticles

**Figure 9:**
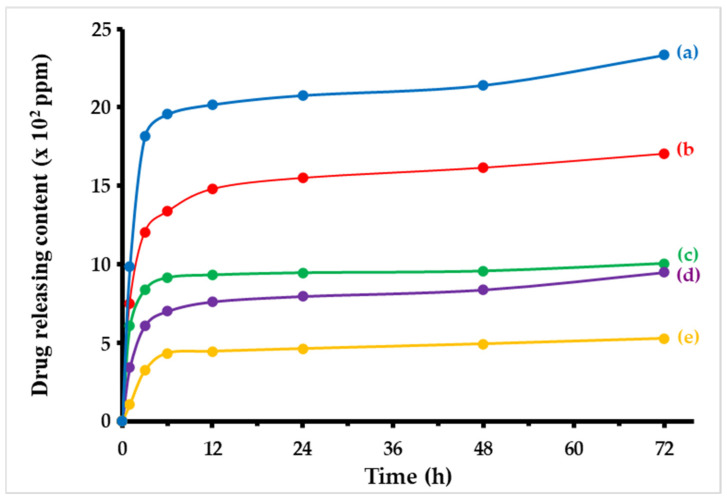
Releasing profiles of blue dextran from the microparticles: native C (a), native SF (b), and C/SF composites at 3:1 (c), 1:1 (d), and 1:3 (*v*/*v*) ratios (e).

The blue dextran release profiles from the prepared microparticles are displayed in Figure 9. According to the results, blue dextran was released quickly during the first few hours of the experiment (1–12 h) and then gradually until 24 h after testing. The C microparticle released the most drug content, followed by the SF microparticle, according to the drug-releasing profiles. The 3:1 mixing ratio released the most drug content of the C/SF composites, followed by the 1:1 ratio and the 1:3 ratio, which released the least. Blue dextran was first released by the native SF microparticle and then by the native C microparticle. According to the results, the composite material significantly outperformed the single material in terms of drug retardation. A water reaction causes the microparticles’ surface texture to burst, releasing the drug-loaded microparticle [35]. In its structure, SF contains high crystalline regions. These areas affected the microparticle’s solid texture, which optimized the drug release and the microparticle’s resistance to water. The drug release profile, which is influenced by various material physical characteristics, is linked to encapsulation efficiency [51]. According to the thermal stability results and release profiles, the hydroxyl groups in the C structure raised the charge on the material’s surfaces, which led to an interaction between the SF’s charge groups and the stability of the composite microparticles. Consequently, compared to both native polymers, the C/SF composite microparticles can shield the release of blue dextran at higher temperatures.

## 4. Conclusions

The SF microparticles were prepared into a spherical shape more easily than the C microparticles. We suggest that the morphological surfaces and shapes of the microparticles are altered according to different parameters. The SEM images revealed that the C/SF composite microparticles could also be constructed; even the mixing ratios are the main factor in giving a suitable target. The C/SF composite with a 1:3 ratio is the suitable composite to receive a satisfactory microparticle. The ATR-FTIR spectra represents the main functional groups in each polymer structure. However, some light absorption peaks were shifted and appeared in the C/SF composite microparticles. This indicated the presence of interactions between the functional groups C and SF. This led to improved thermal stability and increased charred microparticle residue. Given the crystalline structure of both native and composite microparticles, this conclusion was connected to the XRD patterns. To examine the release profile over a 72-h period, blue dextran was loaded into each prepared microparticle. The results obtained demonstrate that the types of prepared microparticles had varying effects on the release of the drug content. The C microparticle released the most drug, whereas the C/SF composite microparticle, at a 1:3 ratio, released the least. This implies that the raw materials used to construct the microparticle, which could be altered by modifying the mixing ratios prior to microparticle preparation, were responsible for the release profile. In conclusion, hydrophilic molecule loading for drug-controlled release applications could be promising for both silk fibroin and cellulose.

## Figures and Tables

**Table 1:** Summary of the maximums of adsorption positions in the FTIR spectra of different microparticles.

Types	Absorption (cm^−1^)				
	O-H Str.	C-H Str.	C=O Str.	N-H Str.	C-O Blend
Native C	3350	2899	1632	-	1018
C/SF (3:1)	3348	2928	1629	1529	1014
C/SF (1:1)	3348	2956	1629	1529	1014
C/SF (1:3)	3348	2956	1629	1529	1014
Native SF	3325	2956	1629	1529	998

**Table 2:** The T*_d, max_* of the prepared microparticles.

Types	T*_d, max_* (°C)	Charred Residue Weight at 800 °C (%)
Native C	150, 310	25
C/SF (3:1)	131, 280, 314	28
C/SF (1:1)	132, 280, 317	30
C/SF (1:3)	138, 280, 328	35
Native SF	260, 303	27

## Data Availability

The original contributions presented in the study are included in the article, further inquiries can be directed to the corresponding author.
